# Autism and the Right to a Hypersensitivity-Friendly Workspace

**DOI:** 10.1093/phe/phab021

**Published:** 2021-08-01

**Authors:** Bouke de Vries

**Affiliations:** Umeå University and KU Leuven

## Abstract

Many individuals on the autism spectrum are hypersensitive to certain sensory stimuli. For this group, as well as for non-autistic individuals with sensory processing disorders, being exposed to e.g. fluorescent lights, perfume odours, and various sounds and noises can be real torment. In this article, I consider the normative implications of such offence for the design of office spaces, which is a topic that has not received any attention from philosophers. After identifying different ways in which the senses of hypersensitive workers might be protected within these spaces, I show that many of such accommodations can be made at reasonable cost, before arguing that doing so ought to be a legal requirement.

## The Challenges of Being Hypersensitive

Virtually all of us encounter things from time to time that we find noisy, smelly or visually off-putting. For most of us, such experiences do not have a major impact on our lives. As scholars such as Joel Feinberg would put it, they offend us without compromising our well-being and ability to function to such a degree as to cause us harm (Feinberg, 1988).

Things are different for those who are hypersensitive to sensory input, i.e. for those who have an overdeveloped capacity for hearing, seeing, smelling, feeling and/or tasting. Many individuals with autism spectrum conditions (ASC) or, as some prefer to refer to themselves, ‘autists’,^1^ fall into this category.^2^ However, not every person with hypersensitivity is also autistic according to several recent studies, which have found that children with a sensory processing disorder (SPD) ‘show atypical sensory behaviours to the same or greater degree as ASC children’ but without displaying the latter’s primary language and social deficits (Owen *et al*., 2013; Chang *et al*., 2014; Reis *et al*., 2017; Tavassoli *et al*., 2018).

Being hypersensitive can, and frequently does, affect individuals in highly negative ways. Temple Grandin, a professor of animal science at Colorado State University, recounts:

When I was little, loud noises were […] a problem, often feeling like a dentist’s drill hitting a nerve. They actually caused pain. I was scared to death of balloons popping, because the sound was like an explosion in my ear. Minor noises that most people can tune out drove me to distraction. When I was in college, my roommate’s hair dryer sounded like a jet plane taking off (Grandin, 2009: 63).

As well as sound, hypersensitive reactions are often triggered by visual stimuli. Consider the following testimony by Lori Sealy, a musician from Mississippi:

My visual experience is […] rather radical. Bright light can be painful — honestly, any light can be painful and I often compensate with sunglasses. I can also get overwhelmed by the sheer amount of imagery that my mind is attempting to process at one time. I take in everything in a panoramic sense — and that sometimes makes it hard for me to focus on the central thing I’m supposed to see (Sealy, 2016).

Still another common form of hypersensitivity involves overreaction to smells. For example, Donna Williams, an Australian writer, recollects how the perfume of one specific woman:

Made the inside of my nose feel like it had been walled up with clay up to my eyebrows. Her perfume burned my lungs; my mouth tasted like I had eaten a bunch of sickly smelling flowers (Williams, 1998: 57).

Exactly how hypersensitive individuals react to these and other sensory stimuli (e.g. ones involving touch and taste) varies. Apart from the fact that there are inter-personal differences (Simpson, 2016), the same hypersensitive individual might respond differently to a given sensory stimulus depending on the context (Bogdashina and Casanova, 2016: 96–99). Still, reactions like the ones just mentioned are common among this group, and might in extreme cases result in a partial or full shutdown of sensory channels, leaving the overstimulated person partially or wholly incapacitated (Bogdashina and Casanova, 2016: 70).

Whereas being hypersensitive can, and usually does, hamper people’s welfare and ability to function within society, then, the question of what moral duties, if any, states have to help protect those with overdeveloped senses has not been investigated by scholars. In this article, I help to fill this lacuna by considering the normative implications of hypersensitivity for the design of office spaces. Doing so is important, as research has shown that a large proportion of autistic employees struggle with sensory overload at work (e.g. Beardon and Edmonds, 2007; Baldwin *et al*., 2014; Lorenz *et al*., 2016; Hayward *et al*., 2018). For example, a report by Beardon and Edmonds (2007) found that, among 237 UK-nationals with Asperger Syndrome (AS) who had filled out a questionnaire about living with AS, over a third reported this problem. To gain a sense of the kinds of sensory issues that these individuals faced, consider some of their comments:

- ‘Hate noise, but endured 10 years in an open plan staffroom, to my acute daily discomfort.’
- ‘I cannot cope with excessive/odd noise—both from colleagues warbling (sorry, I don't mean to be rude) or from high-pitched electronic and similar machinery.’
- ‘Any kind of noise can be annoying some days. All loud noise is painful always.’
- ‘Fluorescent lights make me ill.’
- ‘Sensitive to noise, light, smells etc etc. I need to work in a quiet environment, preferably on my own with as little artificial lighting as possible and no strong odours.’

The remainder of this article is structured as follows. I begin by showing that there are various ways in which employers might accommodate the sensory needs of hypersensitive workers within office spaces. Next, I suggest that many of these accommodations can be made at reasonable cost, before arguing that doing so ought to be legally required.

## Office-Space Accommodations for Hypersensitive Employees: Some Examples

Congruent with the well-known dictum, ‘ought implies can’ (see e.g. Vranas, 2018), I assume in this article that employers should *only be required* to accommodate hypersensitive office-workers if it is possible for them to do so. To show that it is, consider first some of the ways in which they might protect hyper-auditory employees from sensory overload.^3^ One way in which they may do this is by installing carpet flooring or soft flooring, which are less noisy than laminated flooring (National Autistic Society, 2018). Another way is for them to allocate offices to hyper-auditory workers that are not in the vicinity of photocopiers, shredders, printers; and heating, ventilation and air conditioning systems (Gaines *et al*., 2016: 163). Furthermore, when choosing locations for future office buildings, they could seek to avoid locations near railways, busy commercial sites, and roadways with high volumes of traffic insofar as this does not interfere with key organizational objectives, such as being easily reachable by clients (Pedder & Scampton Architects, 2017).

In order to accommodate employees with olfactory hyper sensibilities, some authors have recommended that employers use a background fragrance that drives out the smells of perfumes and deodorants (Clements and Zarkowska, 2000: 80). An alternative measure would be for them to forbid their workers from wearing (strong-smelling) perfume and deodorant. However, since this measure is considerably more intrusive, some might favour the previous measure, possibly combined with a policy that encourages employees to eschew wearing (strong-smelling) perfume and deodorant without forcing them to do so.

Still another set of office-space accommodations addresses the sensory needs of hyper-visual employees. One way in which this may be done is by painting the office walls in low-arousal colours, such as cream and pastel shades (Gaines *et al*., 2016: 61). Another way involves installing non-fluorescent lights within these spaces, as some hypersensitive individuals have such accurate sight that they can perceive a 60-cycle flicker, which might cause them to suffer headaches or worse (Grandin, 2009: 70).

At this point, it ought to be noted that, even in the best-located and best-designed offices, sensory overload is not always avoidable. As such, it can be highly useful for organizations to have a room in which those with overstimulated senses can retreat in order to calm down. Such rooms ought to be low in stimuli, and are ideally used exclusively for this purpose (Simpson, 2016). Alternatively, or in addition, a garden might be used as a place of retreat, as might a tent or a part of a room that is segregated with book cases (Gaines *et al*., 2016: 60).

## The Case for Accommodating Hypersensitive Employees

Having looked at several examples of office-space accommodations for hypersensitive employees, it bears mentioning that, just because this group would benefit from such accommodations does not entail that their employers should be legally required to make them. In order to determine whether this is the case, one must also consider the *costs* of such requirements, which some critics might argue would be excessive.

While there are clearly limits to how much employers or, for that matter, states (see below), can be expected to invest in protecting office-workers from sensory overload, I believe that the current objection is too strong. The easiest way of showing this is to point out the problems with its minor premise. Upon reflection, it turns out that there are various things that employers can do in order to protect their workers from sensory overload at no significant expense. For example, allocating the quietest offices in the building to hypersensitive workers need not cost anything. Likewise, having the office walls painted in cream or pastel shades is not necessarily more expensive than having them painted in bright colours, just as using LED lighting is not necessarily more expensive than using fluorescent lighting.^4^

But even when certain ways of accommodating the sensory needs of hypersensitive office-workers impose costs on the short-term—think, for instance, of a case where an employer replaces the laminate flooring of an office building with carpet flooring even though it could have been used a few years longer—these accommodations will sometimes *repay themselves over time.* One reason for this is that offices that are low in stimuli have been found to have a tendency to increase general productivity (see the studies cited in Al Horr *et al*., 2016: 383 and Kamarulzaman *et al.*, 2011: 265), which may not just be because they make hypersensitive workers more productive, but also because they might enhance the output of other workers, including that of individuals who are *highly sensitive* and who are thought to make up approximately 15–20 per cent of the population (Boterberg and Warreyn, 2016). Another reason is that hypersensitivity-friendly organizations are more likely to attract, as well as to retain, high-functioning autistic workers. Since these workers generally have greater-than-average abilities to detect patterns, remember large amounts of information and concentrate on repetitive tasks (Scott *et al*., 2017; Solomon, 2020), which come on top of relatively high levels of trustworthiness and integrity (Scott *et al*., 2017), many of them are especially qualified to perform monotonous jobs that require high levels of accuracy such as coding and laboratory work (Hagner and Cooney, 2005; Solomon, 2020).^5^

What about cases where (certain ways of) accommodating the sensory needs of hypersensitive office-workers *do impose significant costs* on employers on both the short-term and long-term? Some might say that it would be problematic for this group to incur such costs, or simply to do so above a certain threshold. Apart from the fact that it might hinder competition by creating additional barriers for people to start small businesses, they may argue that *everyone in society* has a duty of justice to help ensure that fellow citizens and residents have fair opportunities for societal participation (cf. Anderson, 1999; Rawls, 1999; Mason, 2006), including fair opportunities for participation in the labour market (Brown, 2021), and that, because of this, the costs of accommodating hypersensitive office-workers should be partially, if not fully, covered by public subsidies.^6^

I will not try to settle here how, if at all, the costs of reasonable accommodations for hypersensitive workers ought to be divided between employers and the state, which is an issue that is well beyond this article’s scope (for a discussion of it, see e.g. Moss and Malin, 1998). The point that I want to make is more modest, namely that *even when* the costs of (some) office-space accommodations for hypersensitive workers are unlikely to be completely off-set over time, there are good grounds for thinking that the accommodations should be made nonetheless when the costs of doing so are not too great (whether this is the case will depend on many factors that I cannot begin to discuss in this article, including on whether the costs in question are absorbed by the employer, the state, or both, and, in case of the employer, on how large the organization is). To see this, notice that, although there are other valuable goods on which this money could be spent—e.g. employers might invest it in offering better services or in making better products, whereas states might use it to alleviate child poverty or reduce carbon emissions, or simply leave the money for tax-payers to spend as they see fit—accommodating the sensory needs of hypersensitive workers is of great moral importance. One reason for this was mentioned when I noted that, for many hypersensitive individuals, working in a hypersensitivity-unfriendly environment takes a heavy toll of their health and welfare. In addition to being problematic in itself, especially given the large amounts of time that people spent on the job (for example, some estimates suggest that Americans spend 25 per cent of their lives at work; Warr and Clapperton, 2009), these harms may render it difficult for them to stay in employment^7^ and thereby threaten their access to various work-related goods. One might think here not only of remuneration and employment-based health insurance, but also of a professional identity that can imbue their life with meaning and structure (Gheaus and Herzog, 2016) and of regular social interaction, which, apart from being valuable in its own right, has been shown to be a petri dish for the development of friendships (e.g. Sias and Cahill, 1998).

## Policy Implications

My aim in this final section is to consider in some detail the policy implications of my arguments thus far. The most important implication is that in countries where there are currently no legal requirements to accommodate hypersensitive workers, legislation ought to be introduced that mandates such accommodations when they can be made at reasonable cost. As far as I am aware, no such legislation currently exists. There are, to be sure, countries where employers have legal obligations to accommodate *autistic employees*, who it was noted in the first section often suffer from one or more forms of hypersensitivity. In the US, the Rehabilitation Act of 1973 and the Americans with Disabilities Act of 1990 require governmental organizations on both the federal level and state level to provide reasonable accommodations to people with disabilities, including to autistic people (Hensel, 2017), insofar as this renders these individuals qualified to do specific jobs. In Europe, both the EU and several of its individual member states have ratified the UN Convention on the Rights of Persons with Disabilities of 2008, which gives people with autism and other disabilities ‘the right to employment in inclusive settings and the right to reasonable accommodation and support to enable them to work effectively’.^8^ However, none of these documents recognize a right to workspace accommodations for non-autistic individuals with SPD, as SPD is not currently recognized as a stand-alone disability by medical authorities such as the American Psychiatric Association (Reis *et al*., 2017).^9^

Besides failing to accommodate people with SPD, there is another way in which existing disability laws and acts fall short of the legal provisions that I am advocating. This shortcoming consist of the fact that they require that reasonable accommodations be made *only once* autistic workers have either informed their employer of their autism and of any special needs that might come with it, including ones for special protections from sensory overload, or demonstrated such needs through their behaviour and functioning (Hensel, 2017: 94–95). There are two problems with this approach as compared to one that requires employers to make certain accommodations for hypersensitive workers *regardless of* whether they currently have such workers or know to have such workers, about which more shortly.

One is that some individuals never tell their employer about their autism, which may leave the latter ignorant of any autism-related special needs that they might have. Reviewing the literature on workplace accommodations for people on the spectrum, Lindsay *et al*. (2021) found that, among four studies that included rates of workplace disclosure, between 25 per cent and 69 per cent of autistic employees had not disclosed their autism to their employer, which, in spite of the small sample sizes, points to a real problem. In most cases, this reluctance was motivated by fears of being stigmatized and of suffering discrimination (e.g. Morris *et al.*, 2015), which causes some autists to try to hide their condition by mimicking the behaviours of their neurotypical peers (Lindsay *et al.*, 2019).

The other problem with the current approach is to do with the fact that decisions about the locations of office buildings, as well as ones about their floor covering, lighting, and so on, have a big impact on how hypersensitivity-friendly a workplace is, but are difficult and/or costly to overturn once implemented. Because of this, it is highly important that certain accommodations be made *even before* hypersensitive individuals have joined the workforce or are known to have done so, which is something that legislation can help to achieve. For although the precise accommodations that hypersensitive workers need will vary from one hypersensitive worker to the next, we have seen in the first section that there are various office-related accommodations—e.g. installing LED lighting rather than fluorescent lighting, ensuring that the walls have cream or pastel colours rather than bright colours—that will help to protect many, if not most, of these individuals from sensory overload.^10^

At this point, a critic might argue that, whether reasonable accommodations for hypersensitive office-workers ought to be legally mandatory will depend on whether there are less restrictive (i.e. more freedom-respecting) alternatives available, and add that such alternatives exist. On this view, states could simply use media campaigns in order to encourage employers to make their offices more hospitable towards (future) workers with overdeveloped senses, and possible offer them subsidies for doing so.

My response is that, while such measures might suffice in a more ideal world, this is unlikely to be the case within contemporary societies. For one thing, the fact that hypersensitivity is a largely invisible and unknown disability makes it reasonable to expect that, *even if* media campaigns were launched to raise awareness of it, a proportion of employers would remain ignorant of its existence and of the ways in which hypersensitive office-workers could be accommodated. For another, simply having this knowledge by no means guarantees that employers will make (enough of) such accommodations, as many may fear that spending money on this will make their organization less competitive. But if these concerns are warranted, then notwithstanding the strong presumptive reasons against the use of state coercion (e.g. Gaus, 1996), it does seem that legislation mandating reasonable accommodations for hypersensitive office-workers is urgently needed.

## Footnotes

1 This preference is usually based on the belief that their ASC should be seen as a valuable part of their identity rather than as a disorder or disease as the term ‘people with autism’ might suggest. For further discussion, see Baron-Cohen (2017) and Jaarsma and Welin (2012).

2 While rates for autistic adults are difficult to come by, Attwood and Wing (1997: 129) estimate that 40 per cent of children with autism have some form of sensory hypersensitivity.

3 For more comprehensive and detailed overviews of ways in which the sensory needs of hypersensitive workers may be accommodated, see e.g. Simpson (2016) and Gaines *et al*. (2016).

4 In fact, some types of LED lamps are cheaper on the long-run.

5 Which helps to explain why technology companies such as Microsoft, Vodafone, SAP, and Hewlett-Packard Enterprise have started to actively recruit autistic workers in recent years (Alsop, 2016).

6 An example of such subsidies may be found among the Disabled Access Credit in the US, which provides credit for small businesses to cover expenditures for the ‘purpose of providing access to persons with disabilities’ (IRS, 2020).

7 And it is noteworthy that no less than 76–90 per cent of autists are estimated to be unemployed across the European Union compared to 11.5 per cent of the general population (Autism Europe, 2014).

8 A similar right is recognized under the revised version of the European Social Charter of 1996, which requires state parties to ‘take adequate measures for the placing of disabled persons in employment, such as specialized placing services, facilities for sheltered employment and measures to encourage employers to admit disabled persons to employment’.

9 Which publishes the influential Diagnostic and Statistical Manual of Mental Disorders (DSM).

10 Among other possible groups, such as employees who are highly sensitive. See the previous section.
